# Comparative Study of the Long-Term Impact of the COVID-19 Pandemic on Mental Health and Nutritional Practices Among International Elite and Sub-Elite Athletes: A Sample of 1420 Participants from 14 Countries

**DOI:** 10.1186/s40798-023-00653-w

**Published:** 2023-11-08

**Authors:** Morteza Taheri, Helmi Ben Saad, Jad Adrian Washif, Luis Felipe Reynoso-Sánchez, Masoud Mirmoezzi, Leila Youzbashi, Khaled Trabelsi, Mozhgan Moshtagh, Hussein Muñoz-Helú, Leonardo Jose Mataruna-Dos-Santos, Ali Seghatoleslami, Farnaz Torabi, Yusuf Soylu, Cem Kurt, Rodrigo Luiz Vancini, Shabnam Delkash, Marjan Sadat Rezaei, Mahdi Ashouri, Shazia Tahira, Mansour Sayyah, Hamdi Chtourou, Ismail Dergaa, Jana Strahler, Andressa Fontes Guimarães-Mataruna, Tyler W. Lebaron, Ebrahim Shaabani Ezdini, Ardeshir Alizade, Hassane Zouhal, Alexander T. Tarnava, Cain Clark, Nooshin Bigdeli, Achraf Ammar, Özgür Eken, Karim Ben Ayed, Nicola Luigi Bragazzi, Hadi Nobari, Mabliny Thuany, Katja Weiss, Beat Knechtle, Khadijeh Irandoust

**Affiliations:** 1 Department of Behavioral and Cognitive Sciences in Sports, University of Tehran, Tehran, Iran; 2 Faculty of Medicine, Research Laboratory “Heart Failure, LR12SP09”, Farhat HACHED Hospital, University of Sousse, Sousse, Tunisia; 3 Sports Performance Division, Institut Sukan Negara Malaysia (National Sports Institute of Malaysia), Kuala Lumpur, Malaysia; 4 Department of Social Sciences and Humanities, Autonomous University of Occident, Los Mochis, Mexico; 5 Faculty of Physical Education and Sport Science, Islamic Azad University, Tehran, Iran; 6 Department of Sport Science, Faculty of Humanities, University of Zanjan, Zanjan, Iran; 7 Research Laboratory: Education, Motricité, Sport et Santé, EM2S, LR19JS01, High Institute of Sport and Physical Education of Sfax, University of Sfax, Sfax, Tunisia; 8 Social Determinants of Health Research Center, Faculty of Health, Birjand University of Medical Sciences, Birjand, Iran; 9 Department of Economic-Administrative Sciences, Autonomous University of Occident, Los Mochis, Mexico; 10 Sport Management Department, Faculty of Management, Canadian University Dubai, City Walk, Dubai, United Arab Emirates; 11 Faculty of Sports Science, University of Birjand, Birjand, Iran; 12 Payame Noor University, Tehran, Iran; 13 Faculty of Sport Sciences, Tokat Gaziosmanpasa University, Tokat, Türkiye; 14 Kirkpinar Sport Sciences Faculty, Trakya University, Edirne, Turkey; 15 Center for Physical Education and Sports, Federal University of Espírito Santo, Vitória, Espírito Santo, Brazil; 16 Department of Sport Sciences, Imam Khomeini International University, Qazvin, Iran; 17 Department of Sports Physiology, University of Mazandran, Babolsar, Iran; 18 Department of Psychology, Virtual University of Pakistan, Lahore, Pakistan; 19 Trauma Research Center, Kashan University of Medical Sciences, Kashan, Iran; 20 High Institute of Sport and Physical Education of Sfax, University of Sfax, Sfax, Tunisia; 21 Primary Health Care Corporation, Doha, Qatar; 22 Research Unit Physical Activity, Sport, and Health, (UR18JS01), National Observatory of Sport, Tunis, Tunisia; 23 Sportpsychology, Department of Sport and Sport Science, University of Freiburg, Freiburg, Germany; 24 Department of Communication and Arts, University of Beira Interior, Covilha, Portugal; 25 Department of Kinesiology and Outdoor Recreation, Southern Utah University, Cedar City, UT USA; 26 Molecular Hydrogen Institute, Enoch, UT USA; 27 University of Zanjan, Zanjan, Iran; 28 School of Medicine, Qazvin University of Medical Sciences, Qazvin, Iran; 29 Laboratoire Mouvement, Sport, Santé, University Rennes, Rennes, France; 30 Institute International Des Sciences du Sport, Irodouer, France; 31 Natural Wellness Now Health Products Inc., Maple Ridge, BC Canada; 32 Faculty of Health, Education, and Life Sciences, Birmingham City University, Birmingham, UK; 33 Control Engineering Department, Faculty of Technical and Engineering, Imam Khomeini International University, Qazvin, Iran; 34 Department of Training and Movement Science, Institute of Sport Science, Johannes Gutenberg-University Mainz, Mainz, Germany; 35 Interdisciplinary Laboratory in Neurosciences, Physiology and Psychology: Physical Activity, Health and Learning (LINP2), UFR STAPS, UPL, Paris Nanterre University, Nanterre, France; 36 Research Laboratory, Molecular Bases of Human Pathology, LR19ES13, Faculty of Medicine of Sfax, University of Sfax, Sfax 3029, Tunisia; 37 Department of Physical Education and Sport Teaching, Inonu University, 44000 Malatya, Turkey; 38 High Institute of Sport and Physical Education, Kef. University of Jendouba, El Kef, Tunisia; 39 Sport Sciences, Health and Movement (2SHM) Laboratory, El Kef, Tunisia; 40 Department of Mathematics and Statistics, Laboratory for Industrial and Applied Mathematics (LIAM), York University, Toronto, ON M3J 1P3 Canada; 41 Faculty of Sport Sciences, University of Extremadura, 10003 Cáceres, Spain; 42 Centre of Research, Education, Innovation and Intervention in Sport (CIFI2D), Faculty of Sport, University of Porto, Porto, Portugal; 43 Institute of Primary Care, University of Zurich, Zurich, Switzerland; 44 Medbase St. Gallen Am Vadianplatz, Vadianstrasse 26, 9001 St. Gallen, Switzerland

**Keywords:** Performance, Athlete, Nutrition, Infectious disease, Health

## Abstract

**Background:**

Although several studies have shown that the Coronavirus Disease 2019 (COVID-19) lockdown has had negative impacts on mental health and eating behaviors among the general population and athletes, few studies have examined the long-term effects on elite and sub-elite athletes. The present study aimed to investigate the long-term impact of COVID-19 lockdown on mental health and eating behaviors in elite versus sub-elite athletes two years into the pandemic. A cross-sectional comparative study was conducted between March and April 2022, involving athletes from 14 countries, using a convenient non-probabilistic and snowball sampling method. A total of 1420 athletes (24.5 ± 7.9 years old, 569 elites, 35% women, and 851 sub-elites, 45% women) completed an online survey-based questionnaire. The questionnaire included a sociodemographic survey, information about the COVID-19 pandemic, the Depression, Anxiety and Stress Scale—21 Items (DASS-21) for mental health assessment, and the Rapid Eating Assessment for Participants (REAP-S) for assessing eating behavior**.**

**Results:**

The results showed that compared to sub-elite athletes, elite athletes had lower scores on the DASS-21 (*p* = .001) and its subscales of depression (*p* = .003), anxiety (*p* = .007), and stress (*p* < .001), as well as a lower REAP-S score indicating lower diet quality (*p* = .013).

**Conclusion:**

In conclusion, two years into the pandemic, elite athletes were likelier to have better mental health profiles than sub-elite athletes but surprisingly had lower diet quality.

## Key Points

- Elite athletes had better mental health profiles compared to sub-elite athletes, with lower levels of depression, anxiety, and stress.
- Elite athletes reported greater psychological support and perceived themselves as more financially secure during the pandemic than sub-elite athletes do.
- Elite athletes were more likely to have poor eating habits compared to sub-elite athletes.

## Background

The first case of Coronavirus Disease 2019 (COVID-19) was reported in Wuhan, Hubei province, mainland China, on December 12, 2019 [1]. COVID-19 is a highly contagious infectious disease caused by the novel Severe Acute Respiratory Syndrome Coronavirus type 2. Since the population was virtually immunologically naïve to the virus and vaccines were not initially available, preventive public health measures consisted primarily of non-pharmaceutical interventions such as "stay-at-home" directives and lockdowns, which were implemented to halt the spread of the disease [2–4]. The COVID-19 outbreak was considered a global health and social crisis, causing high levels of psychological and social distress among various populations around the world [5–8]. Decisions on the lockdown implementation variated in both duration and severity among countries during the pandemic, making it difficult to conduct comprehensive analyses of the long-term effects of COVID-19 confinement; however, there is a clear understanding that more strict or prolonged measures result in more severe problems in different population spheres, such as mental health [9, 10].

The COVID-19 pandemic and the resulting lockdowns have had a significant impact on the sports community worldwide, with athletes unable to train, travel, or participate in competitions [11, 12]. The closure of training facilities has made it difficult for athletes to adhere to their training plans, and many have had to alter their training practices alone, without the guidance of coaching and support staff [13, 14]. This has had a detrimental impact on the physical and mental well-being of athletes, who have also faced financial difficulties due to the loss of income from canceled events.

The limitations on opportunities for sports and physical activity due to the COVID-19 pandemic have led to an increase in sedentary behavior, such as prolonged sitting and increased screen time. These changes in daily routines have significantly disrupted athletes' athletic routines [15], resulting in reduced motivation to train [12, 16] and affected their ability to perform training exercises appropriately [17]. Furthermore, some athletes may have faced financial difficulties, such as contract loss or sponsorship, which may have worsened their mental health challenges during lockdown periods [18]. The COVID-19-induced lockdown has had a detrimental effect on people's mental health [13]. Mental health is a state of well-being in which an individual can recognize their own abilities, manage everyday stressors, work productively and fruitfully, and contribute to their community [19]. During lockdown, athletes have experienced emotional distress and psychological disorders due to the lack of physical activity, isolation from sports teams, separation from the athletic community, ineffective interactions with coaches, and reduced fans/media support [20].

The uncertainty surrounding the return to sports activities, concerns about contracting COVID-19, worries about recovery if infected, financial difficulties, family conflicts, lack of access to workout facilities, and concerns about future performance can all contribute to mental health problems such as stress, depression, anxiety, and sleep disorders [21, 22]. This is particularly true for individuals who lack adequate coping strategies [23–25]. The decrease in opportunities to participate in sport-related social support networks due to the COVID-19 pandemic may result in feelings of loneliness, as reported by Lippke et al. [26]. This can be particularly challenging for elite athletes, who may experience social isolation and anxiety [25]. The elevated levels of anxiety and stress caused by the pandemic can lead to changes in sleeping habits, such as altered bedtimes, increased screen time, and an increased likelihood of sleep disturbances and disorders [17, 27]. Research has shown that disorders such as stress, anxiety, and sleep disturbances have been linked to a lack of physical activity and workouts [28–30]. Athletes who experience depression may have altered sleeping patterns, lack motivation to stay active, and prefer sedentary behavior during leisure time [16, 20]. Interestingly, athletes have been found to have better mental health compared to non-athletes [31]. Among athletic groups, elite athletes have been reported to have higher levels of self-esteem, mental health, and quality of life relative to sub-elite athletes [32–34]. Furthermore, elite athletes have been found to have better psychological and/or spiritual domains, report higher self-satisfaction and peace of mind, and experience less stress and better biopsychosocial functioning [28, 34, 35]. The COVID-19 lockdown has led to an increase in unhealthy eating behaviors, which can lead to weight gain [27] and cardiometabolic disorders. Elite athletes may be better equipped to maintain healthier dietary practices during the pandemic [36]. In addition, in those who were allowed to return to training activities earlier, faster recovery of fitness and body composition was demonstrated [37]. Factors such as economic and social status, social support, and pre-pandemic mental health and physical fitness levels also play a role in determining health-related behaviors during the pandemic [36]. Although several studies have examined the impact of COVID-19 lockdowns on the mental health and eating habits of elite and sub-elite athletes, few studies have looked at the long-term effects of these lockdowns, two years into the pandemic, on these specific populations. Comparing the experiences of elite and sub-elite athletes may help identify specific risk factors that contribute to the resulting mental health status and eating habits. As many countries have started to ease restrictions and reduce safety procedures, it is important to understand the long-term effects of the pandemic on athletes, and to develop evidence-based strategies for monitoring psychological issues and eating disorders during future lockdowns or similar scenarios.

## Methods

### Participants

The research employed a convenient non-probabilistic and snowball sampling method to conduct a cross-sectional study on athletes who were in or had been in lockdown due to the COVID-19 pandemic. The final sample included 1,420 athletes (41% women) from 1702 athletes who were invited to participate in our study (response rate = 83.43%). As inclusion criteria, at the time when the study was conducted, participants must be at least 18 years of age (mean ± standard deviation [SD]: 24.5 ± 7.9 years), have been under confinement for more than one week, and not to have stopped training for more than 1 week due to injury or illness; while failure to complete the survey was considered as a criterion for elimination. The study includes data from 14 different countries of four continents; Asia: (**1)** Iran, (**2)** Turkey, (**3)** United Arab Emirates; Africa: (**4)** Egypt, (**5)** Tunisia, and (**6)** Algeria; America: (**7)** Mexico, (**8)** Brazil, and (**9)** United States of America; and Europe: (**10)** Spain, (**11)** Portugal, (**12)** England, (**13)** Germany, and (**14)** France. The study included athletes who participate at national and international levels, as well as those who practice sports on a recreational basis. Athletes were classified as elite (**i.e.**, semi-professional, high-competitive and professional level) and sub-elite (**i.e.**, amateur and recreational level) athletes. The characteristics of the sample are presented in Table 1.

**Table 1:** Athlete’s level distribution by country (*n* = 1420)

Country	Sub-elite	Elite	Total
Turkey	69	31	100
United Arab Emirates	40	26	66
Iran	102	84	186
Tunisia	72	59	131
Algeria	53	28	81
Egypt	72	35	107
United States of America	53	29	82
Mexico	69	59	128
Brazil	55	36	91
Portugal	69	56	125
England	58	42	100
Spain	65	29	94
Germany	48	35	83
France	26	20	46
Total	851	569	1420

### Procedures

The data for the study were collected between March and April 2022, two years after the World Health Organization declared the COVID-19 pandemic. The questionnaires were transcribed into an online Google form. The researchers in each of the countries involved in the study distributed the instrument through their contacts in sports organizations and clubs who facilitated access to the athletes. Participants were invited to participate via WhatsApp or email through the link to enter the survey. For every country, a researcher was named as the main contact for response questions from the participants of the study. All participants were required to read the instructions, as well as the informed consent, and indicate their agreement to participate in the research by checking a specific box of the online survey. Athletes took an average of 10 to 15 min to answer the entire survey. Those who skipped sections or did not complete the survey were discarded from the sample. The study was approved by the Ethics Committee of Qazvin University of Medical Sciences (IR.QUMS.REC.1401.311), and athletes completed the survey in their native language or the one they were most familiar with. Sociodemographic and COVID-19-related information was collected, and the questionnaire was translated from English into several languages (e.g., Persian, Arabic, Spanish, Turkish, Portuguese, German, and French) using a back-translation method as suggested by the literature [38]. The study used existing translated versions of the Rapid Eating and Activity Assessment for Patients (REAP-S) and Depression, Anxiety and Stress Scale-21 (DASS-21) questionnaires.

### Instruments

#### Sociodemographic and COVID-19 Information

The study used an ad hoc survey to obtain information about three main dimensions of the participants: individual characteristics (e.g., gender, age, body composition, sports history, and education level), social characteristics (e.g., marital status, family, and household), and COVID-19-related information (e.g., history, vaccine, and mental health support). This information was used to analyze and compare the mental health and eating behaviors among the athletes, as presented in Table 1.

#### Eating Behaviors

The REAP-S survey was used to measure the eating behaviors and nutritional status of the athletes [39]. This self-reported tool consists of one item with a Likert scale of five points to evaluate the willingness to make changes in one's eating habits to be healthier and 15 items with a four-point Likert scale to assess food intake from all food groups and diet-related habits. Scores range from 0 to 39 points, with higher scores indicating healthier dietary behavior characterized by optimal intake of fruits, vegetables, and whole grains and decreased intake of sugary foods, processed meats, and fried foods. The REAP-S is known as a useful cross-cultural questionnaire for assessing eating behavior [40–46]. It has also been applied to athletes and is a reliable tool for evaluating the dietary behaviors of this population [42, 47].

#### Mental Health

To assess the impact of the COVID-19 lockdown on athletes, the DASS-21 [48] was used, which is a scale consisting of 21 items that measure symptoms of depression, anxiety, and stress experienced during the past week. Athletes rated each item on a four-point scale, and the final score of each scale was calculated by multiplying the mean score of the scale by two. The DASS-21 has been validated and translated into multiple languages, and the internal consistency of the subscales has been reported to be high [49]. The scale has also been used with athletes during the beginning of the COVID-19 lockdown. In this study, the internal consistency of the DASS subscales was high, with Cronbach's alphas of 0.94, 0.88, and 0.93 for depression, anxiety, and stress, respectively. Overall, the DASS-21 is a reliable tool for assessing the mental health of athletes during the COVID-19 pandemic and has been validated in various languages and contexts.

### Statistical Analysis

Before conducting statistical analyses, the homogeneity and equality of variances of the data were confirmed through the Kolmogorov–Smirnov and Levene's tests, respectively. The data in tables and text were presented as frequency, percentage, mean, and SD. To compare quantitative data of the elite and sub-elite athletes, an independent t test was used, while a Chi-square independence test was used to compare categorical data. The effect size was also calculated. The level of significance was set at alpha *p* < 0.05. All statistical analyses were performed using SPSS v. 23 (IBM Corporation; Armonk, NY, USA) and Excel spreadsheet (Microsoft Corporation; Redmond, WA).

## Results

**Fig. 1:**
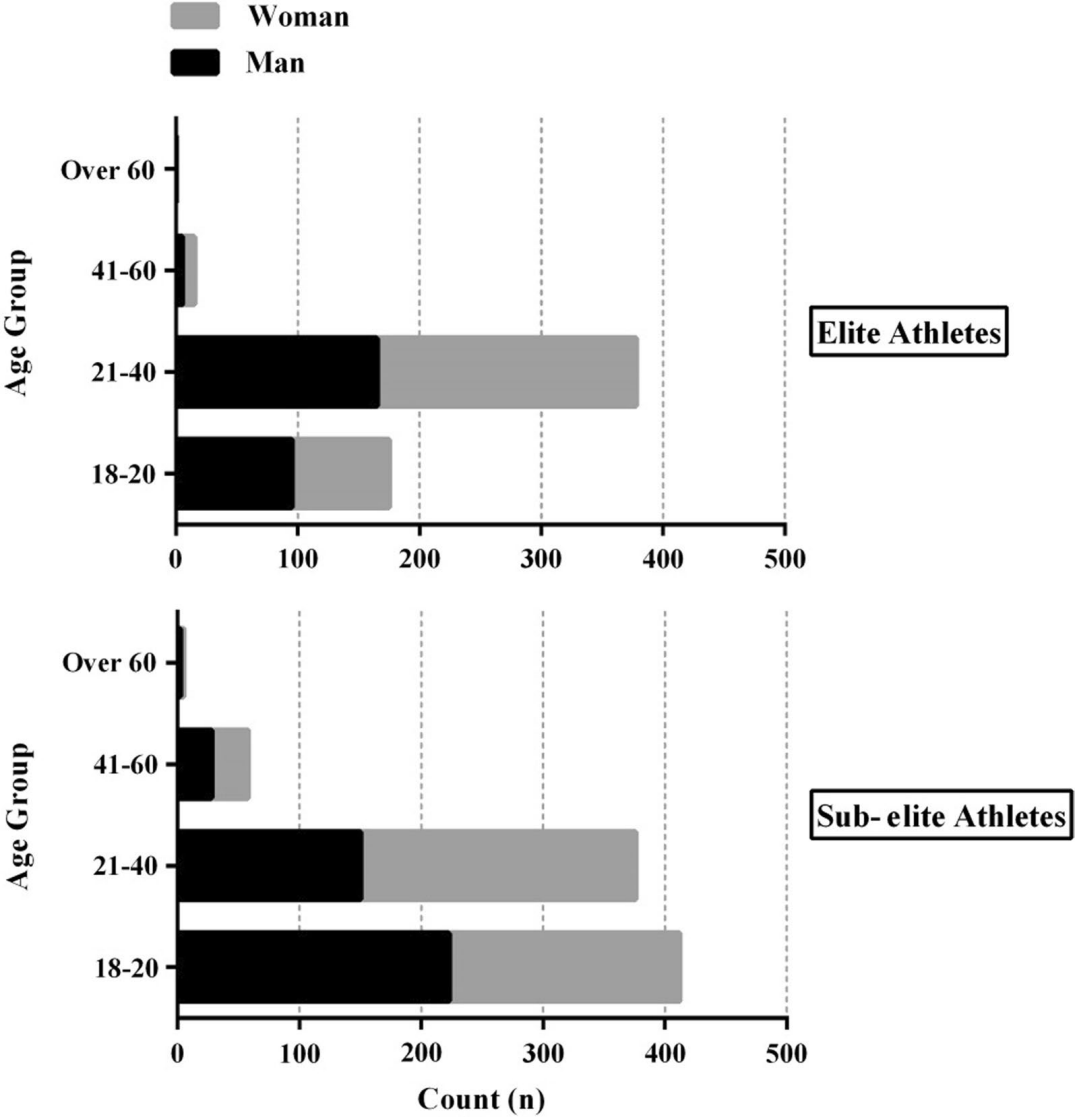
Histogram detailing the gender split and age groups for elite (*n* = 569) and sub-elite (*n* = 851) athletes groups

**Fig. 2:**
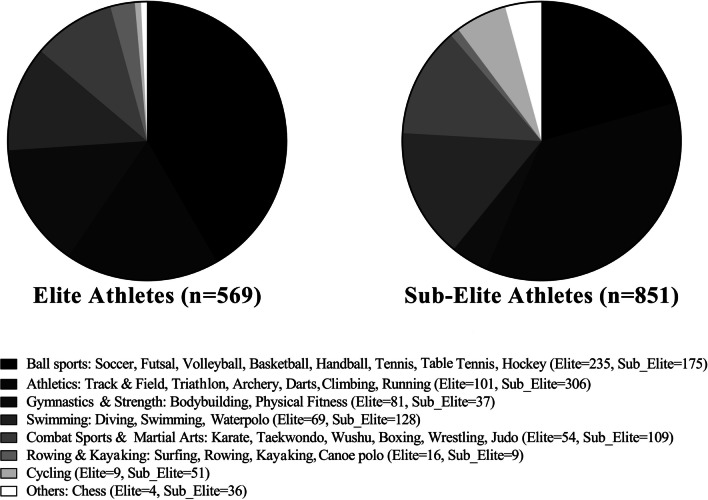
Pie chart of sports played by the elite (*n* = 569) and sub-elite (*n* = 851) athletes’ sample

The distribution of age groups among elite and sub-elite athletes and the type of sport they participated in are shown in Figs. 1 and 2, respectively. Table 1 exposes the athlete’s level distribution by country.

**Fig. 3:**
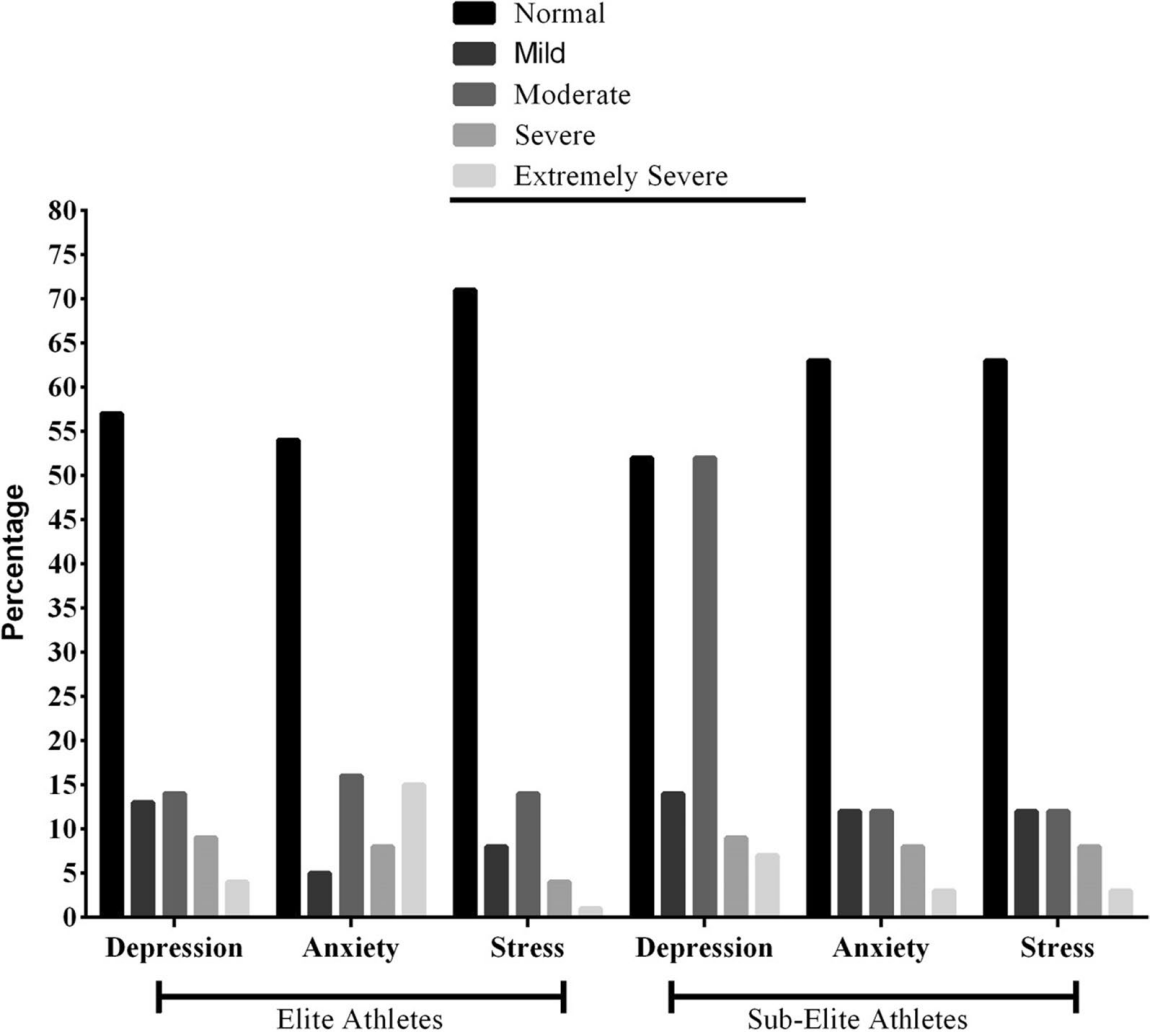
Bar chart of Depression, Anxiety and Stress Scale-21 in elite (*n* = 569) and sub-elite (*n* = 851) athlete groups

Athlete demographics were compared between groups and can be found in Table 2. More than half of the athletes experienced 1 to 4 weeks of social distancing, with sub-elite athletes reporting significantly more social distancing than elite athletes do. The levels of depression, anxiety, and stress were compared between elite and sub-elite athletes, and the results are displayed in Fig. 3. The data suggest that elite athletes had lower levels of depression compared to sub-elite athletes, with 52.1% of elite athletes reporting moderate depression (*p* < 0.001).

**Table 2:** Descriptive statistics of athletes (*n* = 1420)

Demographics	Unit/Category	Sub-elite (*n* = 851)	Elite (*n* = 569)	Total (*n* = 1420)	*p* value
Age^a^	Years	24.2 (8.8)	24.9 (6.6)	24.5 (7.9)	= .756
Height^a^	Cm	171.2 (9.7)	175.6 (10.2)	172.9 (10.2)	= .002*
Weight^a^	kg	69.2 (14.8)	73.3 (17.5)	70.9 (16.1)	< .001*
Sports history^a^	Years	5.8 (7.01)	9.6 (6.3)	7.3 (6.9)	= .022*
Marital status^b^	Solid partnership / Married	166 (19.2)	116 (20.4)	282 (19.9)	< .001*
	Living without a partner / single	685 (80.8)	453 (79.6)	1138 (80.1)	< .001*
Education level^b^	No schooling completed	76 (8.9)	33 (5.8)	109 (7.7)	= .008
	High school graduate	208 (24.4)	132 (23.2)	340 (23.9)	< .001*
	Bachelor’s degree	494 (58.1)	329 (57.8)	823 (57.9)	< .001*
	Master’s degree	52 (6.1)	62 (10.9)	114 (8.1)	< .001*
	Doctoral’s degree	21 (2.5)	13 (2.3)	34 (2.4)	< .001*
Count of households^b^	Live alone	295 (34.7)	193 (33.9)	488 (34.4)	< .001*
	2	88 (10.3)	71 (12.5)	159 (11.2)	< .001*
	More than 2	468 (55.0)	305 (53.6)	773 (54.4)	< .001*
History of COVID-19^b^	Yes	257 (30.2)	219 (38.5)	476 (33.5)	< .001*
Doses of vaccine^b^	0	39 (4.6)	42 (7.4)	81 (5.7)	= .025*
	1	35 (4.1)	35 (6.1)	70 (4.9)	< .001*
	2	403 (47.3)	258 (45.3)	661 (46.5)	< .001*
	3 & more	367 (43.1)	241 (42.3)	608 (42.9)	< .001*
Type of sport^b^	Individual Sport Athlete	398 (46.8)	249 (43.8)	647 (45.6)	< .001*
	Team Sport Athlete	453 (53.2)	320 (56.2)	773 (54.4)	< .001*
Financially secure^b^	Yes	270 (31.7)	203 (35.7)	473 (33.3)	< .001*
Weeks spent social distancing^b^	1–4 Weeks	462 (54.3)	366 (64.3)	828 (58.3)	< .001*
	1–2 Months	179 (21.0)	101 (17.7)	280 (19.7)	< .001*
	2–4 Months	121 (14.2)	73 (12.8)	194 (13.7)	< .001*
	More than 4 Months	89 (10.5)	29 (5.1)	118 (8.3)	< .001*
Sought psychological or mental support: Yes^b^	Before COVID-19	24 (2.8)	9 (1.6)	33 (2.3)	< .001*
	During COVID-19	102 (12.0)	72 (12.7)	174 (12.3)	< .001*

*COVID-19* Coronavirus disease 2019. Data were ^**a**^Mean (standard deviation), ^**b**^Number (%). *p value < .05 (Chi-square independence test or independent t test)

Summary statistics for the DASS-21 and REAP-S in elite and sub-elite athletes are reported in Table 3. There were significant differences in the means of depression (*t*(1418) = 2.99, *p* = 0.003, *η*^2^ = 0.16), anxiety (*t*(1418) = 2.72, *p* = 0.007, *η*^2^ = 0.15), stress (*t*(1418) = 4, *p* < 0.001, *η*^2^ = 0.22), total scale of DASS-21 (*t*(1418) = 3.47, *p* = 0.001, *η*^2^ = 0.19), and REAP-s (*t*(1418) = 2.48, *p* = 0.013, *η*^2^ = 0.13).

**Table 3:** Summary statistics for the DASS-21 and REAP-S in athletes (*n* = 1420)

		Elite (*n* = 569)			Sub-elite (*n* = 851)			Total athletes (*n* = 1420)			*t* (*p* value)	*effect size*
Scale	Subscales	Mean	SD	Range	Mean	SD	Range	Mean	SD	Range		
DASS-21	Depression	8.78	9.37	0–36	10.35	9.88	0–36	9.72	9.71	0–36	2.99*	− 0.48
	Anxiety	8.86	9.31	0–42	10.27	9.75	0–42	9.71	9.60	0–42	2.72*	− 0.43
	Stress	9.89	9.34	0–36	11.99	10.01	0–36	11.15	9.79	0–36	4.0*	− 0.53
	Total scale	27.53	26.15	0–114	32.62	27.61	0–114	30.58	27.2	0–114	3.47*	− 0.54
REAP-S		23.69	7.44	0–28	24.69	7.48	0–39	24.29	7.48	0–39	2.48*	− 0.51

*DASS-21* Depression, Anxiety and Stress Scale-21. *REAP-S* Rapid Eating and Activity Assessment for Patients. *SD* Standard deviation. Scores on the DASS-21 are multiplied by 2 to calculate the final scores. *p value < .05 (independent t tests between Elite and Sub-elite athletes)

## Discussion

This study aimed to explore and compare the impact of the COVID-19 pandemic on the mental health and dietary habits of elite and sub-elite athletes worldwide; two years after the pandemic began. The results showed that elite athletes had better mental health profiles than sub-elite athletes, with lower levels of depression, anxiety, and stress. Elite athletes reported greater psychological support and perceived themselves as more financially secure than sub-elite athletes did during the pandemic. Both groups had good adherence to healthy dietary habits, but elite athletes exhibited less positive eating behaviors than sub-elite athletes did. The results of the study indicate that elite athletes were better equipped to handle the psychological challenges posed by the pandemic but may have been less attentive to their nutritional habits compared to sub-elite athletes. These findings suggest that the better mental health profiles of elite athletes may be attributed to factors such as coping ability, income inequalities, and support.

Elite athletes are often equipped with superior coping abilities, allowing them to navigate the challenges and pressures of competitive sports with greater ease. Research studies support this notion, suggesting that experienced or higher-level athletes tend to have better coping skills, which may enable them to cope more effectively with the uncertainties of the COVID-19 pandemic [25, 50]. Additionally, research has shown that elite athletes generally exhibit better mental health outcomes, with lower levels of stress and depressive symptoms, compared to lower-level athletes [34].

Our study findings align with prior research, suggesting that elite athletes may be more accepting of the negative impacts of the pandemic on their sporting careers, such as the cancelation or postponement of important competitions, including the Olympic Games [13, 51]. Moreover, we found that elite athletes perceived themselves as more financially secure than sub-elite athletes, consistent with research among football players worldwide [52]. Perceptions of financial security have been identified as a significant risk factor for mental health [53–55], which may be particularly relevant for sub-elite athletes who may experience income loss due to sport-related job disruptions [52, 54]. However, this factor may not be as crucial for some elite athletes.

It is worth noting that both elite and sub-elite athletes reported an increase in seeking psychological support during the pandemic, with a higher percentage of elite athletes seeking support than sub-elite athletes. Seeking support was more prevalent among elite athletes than among lower-level athletes during the pandemic or lockdown [12, 14], which is consistent with findings reported by Jaenes Sánchez et al. [51]. Despite the onset of emotional distress as a result of COVID-19, the normalization of seeking support from mental health professionals may have helped elite athletes cope better with the mental health challenges associated with confinement and could prove vital in promoting better coping strategies in the face of future stressful situations.

Studies investigating the impact of COVID-19 on the mental health of athletes have yielded inconclusive results, with some suggesting that athletes are experiencing mental health issues. This underscores the importance of individual-level psychological interventions [34, 52, 54–56] to support athletes in managing their mental health during times of stress and uncertainty. To gain a more comprehensive understanding of these issues, it is crucial to continue researching this phenomenon even after it has been declared a solved or controlled problem.

Based on the DASS-21 results, our study participants exhibited normal to moderate levels of depression, anxiety, and stress. This differs from what has been reported in other studies that found high rates of depression [57] and stress [58] among the general population in Italy. However, some studies conducted at the beginning of the COVID-19 pandemic among athletes found an increase in psychobiological stress response [35], and high levels of dysfunctional psychological responses [35, 50]. Similar findings have been reported in studies carried out more than a year after the pandemic began [55, 56]. This disparity in findings could be attributed to differences in the investigations' timing and the extent of social distancing measures in place at the time of data collection. It is possible that our study participants had less restricted measures of social distancing, which may have enabled them to return to their sports activities more easily than participants in other studies. Our findings are consistent with previous research indicating that increased physical activity has a protective effect on mental health, such as reducing anxiety symptoms associated with the COVID-19 pandemic [51, 59]. It is also possible that our participants underwent a learning process and familiarized themselves with the conditions of the new pandemic world, which may have contributed to their improved mental health outcomes [34, 56].

Contrary to the existing literature, our study found that sub-elite athletes have better dietary habits than elite athletes. A systematic review by Heaney et al. [60] concluded that elite athletes generally have better eating habits than sub-elite or non-athletes. However, a study conducted with Turkish taekwondo athletes [61], as well as research carried out with elite Iranian athletes [62] during the COVID-19 pandemic, reported similar results to our study, where lower-level athletes exhibited more positive nutritional habits. This behavior could be explained by the fact that elite athletes typically have more restricted diets and better nutritional habits during normal circumstances, the COVID-19 pandemic disrupted their routines and made it more challenging for them to adapt to changes in their eating habits. Additionally, the cancelation or modification of competition schedules may have led to increased relaxation and altered eating behavior, such as consuming more food or snacks while spending more time at home.

It is important to provide nutritional support to athletes to minimize the negative impact of COVID-19 on their eating habits through online education or interaction with athletes [20, 63]. Previous research reported how important it is for elite athletes to maintain good eating habits to help their sleep quality during the COVID-19 pandemic [64]. However, there is limited research on the eating behavior of athletes during the pandemic, and it appears that maintaining optimal weight may be an additional stressor for elite athletes. Therefore, further research is needed, especially comparisons between elite and sub-elite athletes, to gain a better understanding of these issues.

Additionally, the REAP-S data collected in our study indicated positive nutritional practices in both groups [41]. Some studies have reported positive changes in eating behaviors during the pandemic [7, 65]. This may be attributed to individuals having more time to prepare their own food, coupled with restrictions on getting food outside the home and a greater awareness of the importance of maintaining a healthy diet [7, 66]. Therefore, our study contributes to understanding how the COVID-19 pandemic has affected the overall food consumption and dietary habits of both elite and sub-elite athletes.

The present study has several strengths, including the availability of a survey in multiple languages that has been widely distributed across various continents. This cross-cultural design involved scientists and researchers from diverse sports science disciplines and countries, who worked together to facilitate the study process. Moreover, this study focused on elite and sub-elite athletes from different continents and countries, and only a few comprehensive studies have been conducted in this field. However, our study does have some limitations. One of the limitations was the failure to consider the influence of sports type (individual vs. team). As participating in a team sport could have a protective effect on mental health, this difference may be a confounding factor. Future studies should take this factor into account and compare the effectiveness of each sport type on athletes' mental health. Additionally, the cross-sectional design of the study was the other limitation. It should also be noted that online surveys commonly suffer from two serious methodological limitations. First, the population to which they are distributed cannot be described, and respondents with biases may select themselves for the sample. We are also aware that some factors, such as coping abilities, psychological support, economic security, and the period of the study, could have influenced the observed results. Differences in the severity of lockdowns experienced in different countries may influence the responses of participants. Furthermore, because lockdown was a “surprise” measure in many countries, we were unable to develop and disseminate the survey "before" lockdown for baseline measures. In this study, one additional limitation could be the reliance on self-reporting by the athletes regarding their experiences during the pandemic. Since a significant amount of time has passed, there may be a possibility of recall bias or inaccuracies in their responses. Furthermore, future research could consider using more objective measures or cross-referencing with other sources of information to validate the athletes' responses.

## Conclusions

This global study investigated the long-term effects of the COVID-19 pandemic on the mental health and nutritional practices of elite and sub-elite athletes. According to the study’s findings, despite the changes in everyday life caused by the COVID-19 pandemic, elite athletes appeared to have better mental health outcomes than sub-elite athletes, as indicated by lower levels of emotional distress as reflected in their DASS-21 score. Furthermore, the data revealed that elite athletes were more likely to have poor eating habits compared to sub-elite athletes, which is an important finding that may be explained by a lack of guidance/education (or even willingness) to maintain their nutritional regimen (e.g., preparing their own food), among others.

## Data Availability

The datasets generated during and/or analyzed during the current study are available from the corresponding author on reasonable request.

## Abbreviations

COVID: Corona Virus Disease
DASS: Depression, Anxiety and Stress Scale
REAP-S: Rapid Eating Assessment for Participants
